# Model verification tools: a computational framework for verification assessment of mechanistic agent-based models

**DOI:** 10.1186/s12859-022-04684-0

**Published:** 2022-05-19

**Authors:** Giulia Russo, Giuseppe Alessandro Parasiliti Palumbo, Marzio Pennisi, Francesco Pappalardo

**Affiliations:** 1 Department of Drug and Health Sciences, University of Catania, 95125 Catania, Italy; 2 Department of Mathematics and Computer Science, University of Catania, 95125 Catania, Italy; 3 Computer Science Institute, DiSIT, University of Eastern Piedmont, 15121 Alessandria, Italy

**Keywords:** Agent-based models, Verification assessment, In silico trials, Medicinal product, Regulatory context, COVID-19

## Abstract

**Background:**

Nowadays, the inception of computer modeling and simulation in life science is a matter of fact. This is one of the reasons why regulatory authorities are open in considering in silico trials evidence for the assessment of safeness and efficacy of medicinal products. In this context, mechanistic Agent-Based Models are increasingly used. Unfortunately, there is still a lack of consensus in the verification assessment of Agent-Based Models for regulatory approval needs. VV&UQ is an ASME standard specifically suited for the verification, validation, and uncertainty quantification of medical devices. However, it can also be adapted for the verification assessment of in silico trials for medicinal products.

**Results:**

Here, we propose a set of automatic tools for the mechanistic Agent-Based Model verification assessment. As a working example, we applied the verification framework to an Agent-Based Model in silico trial used in the COVID-19 context.

**Conclusions:**

Using the described verification computational workflow allows researchers and practitioners to easily perform verification steps to prove Agent-Based Models robustness and correctness that provide strong evidence for further regulatory requirements.

## Background

The recent openness from both European and USA Regulatory Agencies [1, 2] to the possibility of using computer modeling and simulation for providing some of the regulatory evidence needed for the assessment of *safeness* (i.e., when it does not worsen the health of the recipient) and *efficacy* (when it does improve the recipient’s health) of novel medical compounds has paved the way to the application of the so-called *in-silico* trials. Computational simulations can be used to strengthen, or to possibly substitute, the results coming from experiments involving cell cultures and animals (i.e., *pre-clinical trials*) before and human volunteers (i.e., phase I, II, III, and IV *clinical trials*) then.

While the regulatory protocol for the assessment of safeness and efficacy (i.e., *qualification*) of a medical product is well established when classical clinical trials are considered [3], there is still a lack of a common consensus [4–7] on how to assess the “credibility” of a computational model. Even if verification and validation techniques can be in general borrowed from other research fields (i.e., statistics, engineering, mathematics, and physics), it has been mandatory to establish which steps must be carried on and which methodologies must be used on each of them to *qualify,* through verification, validation, and uncertainty quantification (VV&UQ) procedures, any computational model to be used for In Silico Trials (ISTs).

To date, few model credibility standards and approaches have been discussed [8–10]. In the field of In Silico Trials, Viceconti et al. [11] proposed a theoretical framing for the problem of assessing the credibility of a predictive model for ISTs that considers the epistemic specificity of the research field and is general enough to be used for different types of models, including simulators based on Agent-Based Models (ABMs), that have become increasingly popular in this scenario.

Thanks to ABMs ability to readily describe complicated biological behaviors, laws, and interactions involving cells and molecules without the need for complex mathematical formulas, these models are increasingly applied to simulate human pathophysiology. Specifically, AMBs are useful to predict disease progression and related response to various treatment administrations, or in specific conditions where the immune system involvement is considered, and also to assist in discovering and developing novel vaccines.

In ABMs, entities (also called agents) are tracked individually, and interactions are recorded one by one, allowing for the inference of the system global emergent behavior as the sum of the agents individual behaviors (bottom-up approach). As ABMs lack a strong mathematical formalization and a standard verification process, some verification steps must be refined and customized better to meet their characteristics [12, 13]. In this scenario, Curreli et al. [14] adapted the theoretical framework mentioned above for assessing the credibility of an ABM simulator of the immune system in the presence of tuberculosis disease. However, the numerical and statistical procedures carried on the verification procedure have been executed manually, using different tools and software.

To facilitate the work of researchers employed in the development of computational models for ISTs and to speed up the verification procedure, we developed “Model Verification Tools” (MVT), a suite of tools based on the same theoretical framework described above and with a user-friendly interface for the evaluation of the deterministic verification of discrete-time models, with a particular focus on agent-based approaches. The toolkit makes it simple for researchers to check many parts of models for possible flaws and inconsistencies that could influence their outcomes.

## Implementation

### The verification workflow and its application to ABMs

Curreli et al. proposed a theoretical framework that aims at defining the steps for assessing the credibility of mechanistic models used in the context of in-silico trials for medicinal products [14]. Here, we recall such a verification workflow, as it represents the starting point for MVT development. The workflow considers two verification procedures that can be carried out independently, i.e., deterministic and stochastic model verification. ABMs usually make use of pseudo-random number generators initialized with different random seeds for reproducing different stochastic behaviors. Keeping constant or varying the random seed over a set of simulations, it is then possible to analyze the model behavior from a deterministic or stochastic point of view. Hence, stand-alone procedures for the deterministic and stochastic verification procedures can be provided.

For the deterministic model verification, the workflow takes into consideration the following steps:

1. Existence and uniqueness analysis
2. Time Step Convergence Analysis
3. Smoothness Analysis
4. Parameter sweep analysis

Moreover, for stochastic model verification, the following steps are also considered:

5. Consistency
6. Sample Size.

At present, MVT includes the analysis tools for steps 1–4, as these represent the most important ones. Steps 5 and 6 will be implemented in the next release of the tool.

The existence procedure checks for solution existence in the acceptable range of the input parameters. Uniqueness focuses on checking for possible numerical and discretizations (i.e., round-off errors) due to the limited numerical precision of computing platforms that may influence solution results over different runs executed with the same seed. While existence can be checked by assuring that the computational model returns an output value for a given reasonable input range, uniqueness can be verified by checking that identical input sets always entitle the same outputs with, at most, a minimal tolerated variation determined by the used numerical rounding algorithm.

Time step convergence analysis aims at assuring that the time approximation introduced by the Fixed Increment Time Advance (FITA) approach used by most ABM frameworks and tools does not extensively influence the quality of the solution. The same model is run with different time-step lengths to calculate the percentage discretization error according to the following equation:

$$e_{q}^{i} = \frac{{q^{i*} - q^{i} }}{{q^{i*} }}*100$$

where *q*^i*^ is an output reference quantity (i.e., the peak value of the simulation, final value or mean value) obtained by the simulation executed at the smallest reference time-step that maintains the execution of the model still computationally tractable (i*); *q*^*i*^ represents the same output reference quantity obtained with a time-step *i* (with *i* > i*), and *e*_*q*_^*i*^ is the percentage discretization error. In their work, Curreli et al. proposed to assume that the model converges if the error *e*_*q*_^*i*^ < 5%.

Smoothness analysis was proposed to calculate the smoothness of the solution, bearing in mind that possible errors in the numerical solution may lead to singularities, discontinuities, and buckling. The coefficient of variation D is computed as the standard deviation of the first difference of the time series scaled by the absolute value of their mean for all the output time series to evaluate the smoothness. To this end, a moving window is used, and thus for each time observation y_t_ in the output time series, the k nearest neighbors are considered in the window: y^k^_t_ = {y_t-k_, y_t-k + 1_,…, y_t_, y_t + 1_,…, y_t + k_}. Currelli et al. used k = 3. The higher D is, the higher is the risk of stiffness, singularities, and discontinuities.

Finally, parameter sweep analysis is used to assure that the computational model is not numerically ill-conditioned. In general, the procedure involves sampling the entire input parameter space to check if for particular input sets, the model fails to produce a valid solution or if the solution is valid but outside the expected validity range. Furthermore, by introducing slight variations on the input values, the analysis can be used to verify if such slight variations entitle significant variations on the output values, suggesting an abnormal sensitivity to some inputs.

While in their paper Curreli et al. proceed by using a two-step procedure for reducing the input set size first, and to check the effects of the most relevant selected inputs on the outputs then, we believe that similar results can be obtained by using well-known standard stochastic sensitivity analyses, such as variance based (Sobol) sensitivity analysis or Latin Hypercube Sampling-Partial Rank Correlation Coefficient (LHS-PRCC), which have been then introduced inside MVT. The latter, in particular, uses a Latin Hypercube Sampling (LHS) over the entire input parameter range to calculate the Partial Rank Correlation Coefficient (PRCC) values between the input values and the selected output value. In this way, it is possible to estimate the influence that any input parameter has on the output value, independently from the variation carried over the other input parameters from a stochastic point of view. This procedure can also be carried at any time point to check the influence of the inputs on the output over time. LHS-PRCC is a robust sensitivity analysis technique for nonlinear but monotonic relationships between inputs and output.

### Model verification tools

**Fig. 1:**
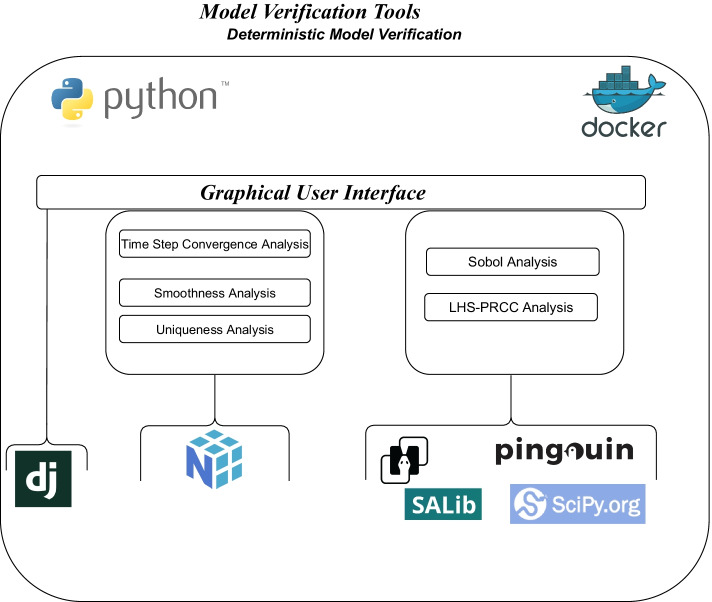
Components and libraries of model verification tools

Model Verification Tools (MVT) is an open-source tool^1^ that offers helpful analysis to verify discrete-time stochastic simulation models. Figure 1 shows the architecture, the software, and the libraries used to develop MVT. The tool is fully developed using Python 3.9 programming language [15], the Django^2^ environment to create the web infrastructure, and Docker.^3^ Thanks to this last component, we were able to build up a stand-alone software platform (a docker container) that can be used on any operating system. This represents a huge leap ahead in respect to its preliminary web-based implementation [16]. This version brings several improvements, among which a considerable reduction of the latency times related to large file uploading and the possibility of taking full advantage of the system resources for more complex analyses. Among the libraries used for Uncertainty and Sensitivity Analysis, “Pingouin” [17], “Scikit'' [18] and “Scipy” [19] were used to perform the LHS-PRCC analysis, while the library “SALib” [20] was chosen to perform the Sobol sensitivity analysis.

**Fig. 2:**
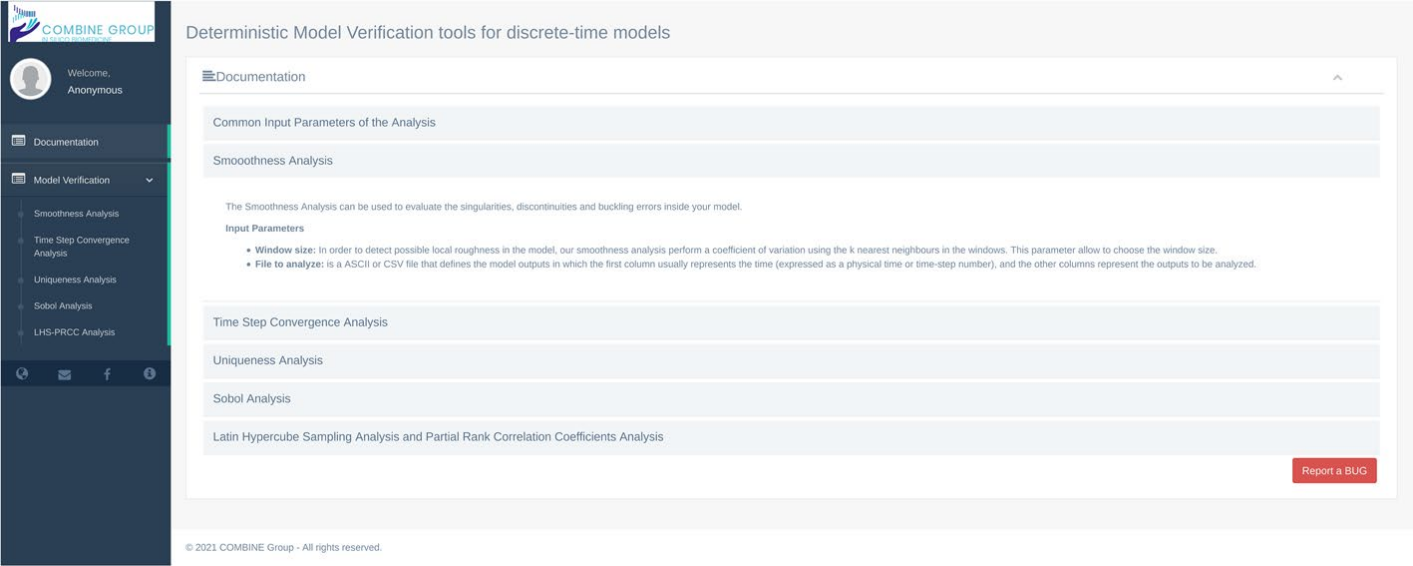
The documentation page of MVT

Regarding the libraries used for the deterministic model verification techniques, we used “Numpy” [21], the fundamental python package for scientific computing. The Graphical User Interface (GUI) of MVT (Fig. 2) consists of two main menus: 1) Documentation and 2) Model Verification. The documentation menu gives a brief description of each technique and explains in detail each input parameter.

The second menu consists of five sub-menu: i) Smoothness Analysis, ii) Time step Convergence Analysis, iii) Uniqueness Analysis, iv) Sobol Analysis, and v) LHS-PRCC Analysis.

#### Smoothness analysis

**Fig. 3:**
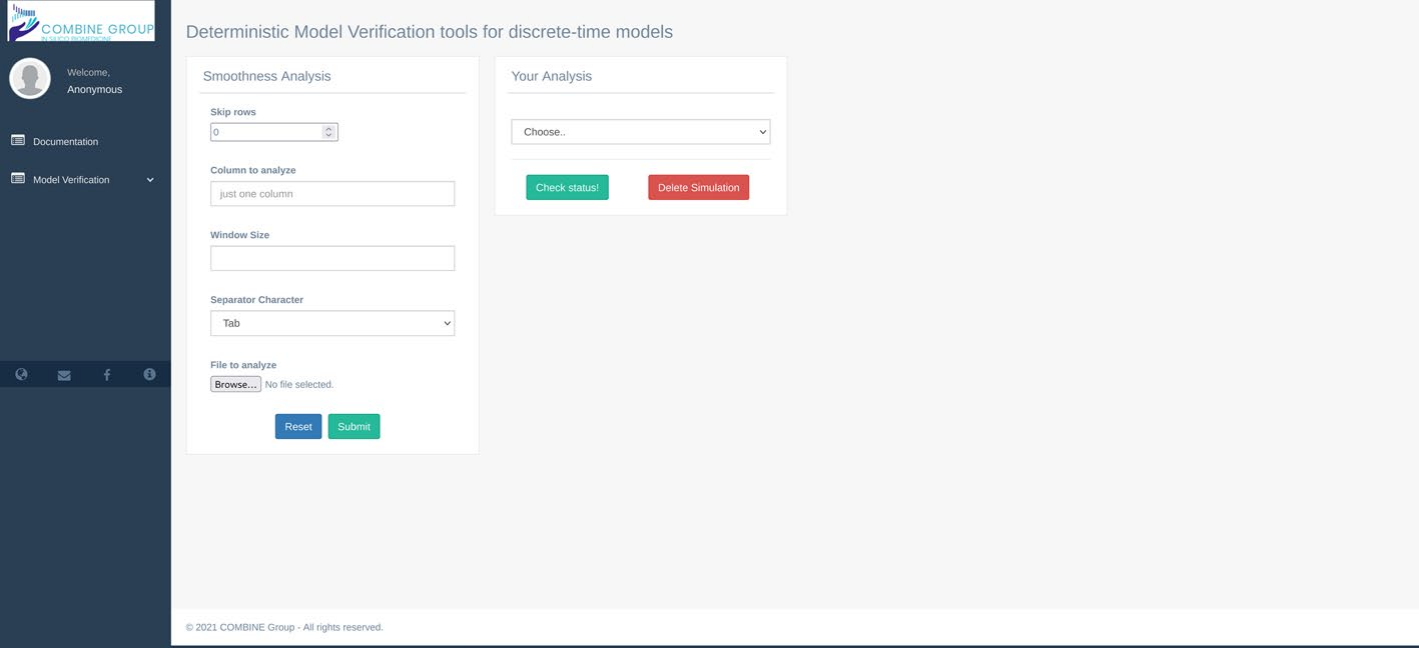
The smoothness analysis GUI. The box on the left side represents the list of parameters to perform the analysis; on the right side, the “Your Analysis” box contains the list of the completed results analysis

The model may suffer from singularities, discontinuities, and buckling errors. The Smoothness Analysis allows detecting these errors. To perform this analysis, a setting up of the following parameters is required (Fig. 3): i) “Skip rows” panel allows the ignore specific rows from the analysis, for example, header lines of the input/output files that have to be removed; ii) “Column to analyze” panel allows the selection of the output column to be analyzed from the input/output file uploaded by the user; iii) “Window Size” panel allows to define the size of the window, i.e., the choice of the k nearest neighbors for the analysis; iv) “Separator character” panel allows defining the correct separator character of the input files (i.e., comma, space, tab); v) “File to analyze” panel allows the user to upload a CSV or ASCII file defining the output file on which to perform the analysis. After clicking on the submit button, the analysis applies the procedure described above on the column selected by the user. Then, by choosing the listed results on the box “your analysis” reported on the right side of Fig. 3, the user can look at the analysis results and the related produced plots.

#### Time step convergence analysis

**Fig. 4:**
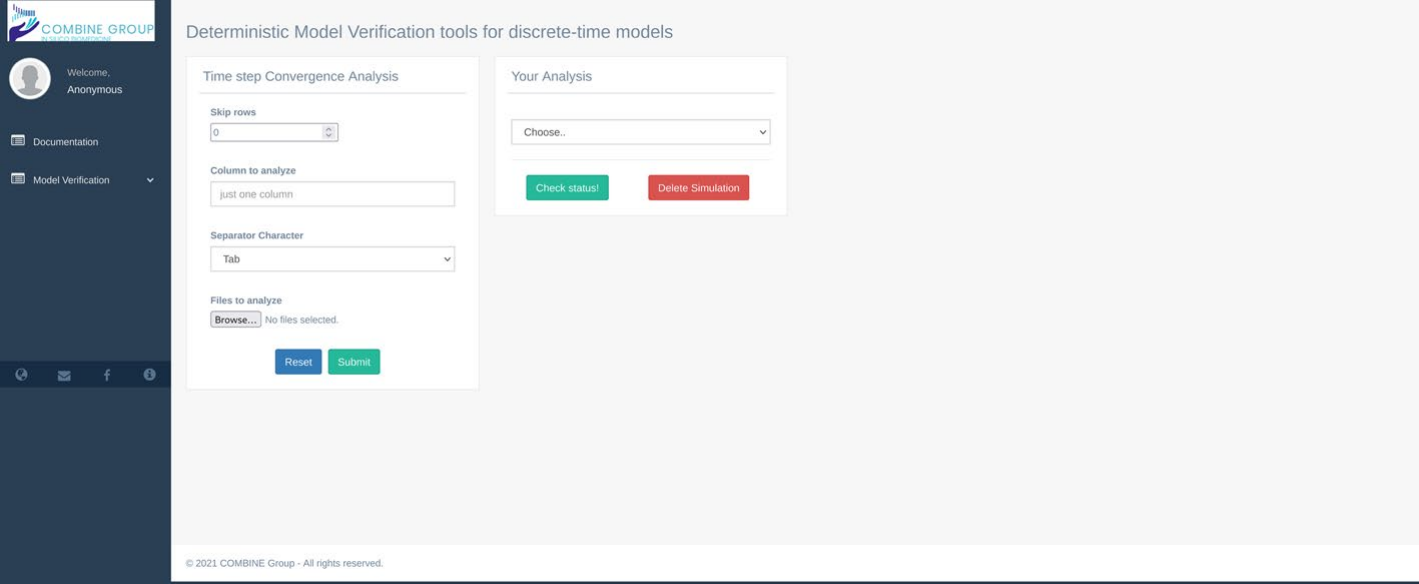
The Time step convergence analysis GUI. The box on the left side represents the list of parameters to perform the analysis; on the right side, the “Your Analysis” box contains the list of the completed results analysis

Time step Convergence Analysis allows one to determine if the model behavior converges as the time-step length becomes narrower. In this version of MVT, the numerical measures considered to evaluate the global convergence of the model are the maximum value achieved throughout the simulation (Peak Value—PV), the time-to-peak-value, the final value (FV), the Pearson Correlation Coefficient (PCC), and the root-mean-square error (RMSE). To perform the study of the model convergence, it is essentially setting up the parameters from the interface (Fig. 4). This analysis essentially takes into account the same parameters described for the smoothness analysis. After clicking on the submit button, the user can retrieve the plots of the measures mentioned above produced by the algorithm in the box “your analysis” shown on the right side of Fig. 4.

#### Uniqueness analysis

**Fig. 5:**
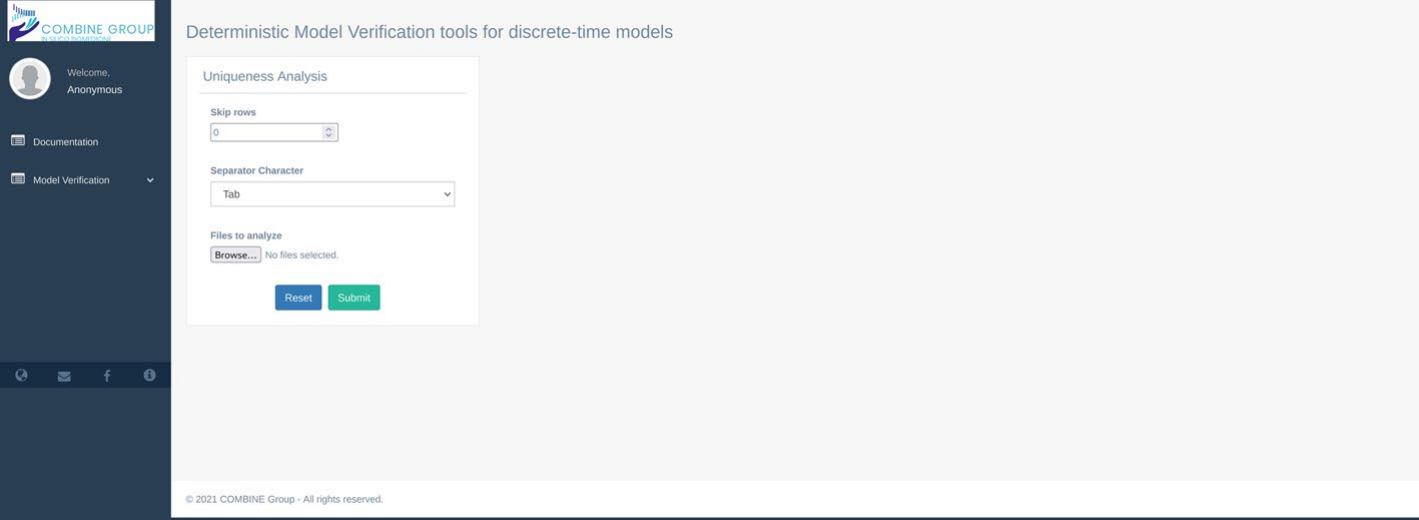
The uniqueness analysis GUI

**Fig. 6:**
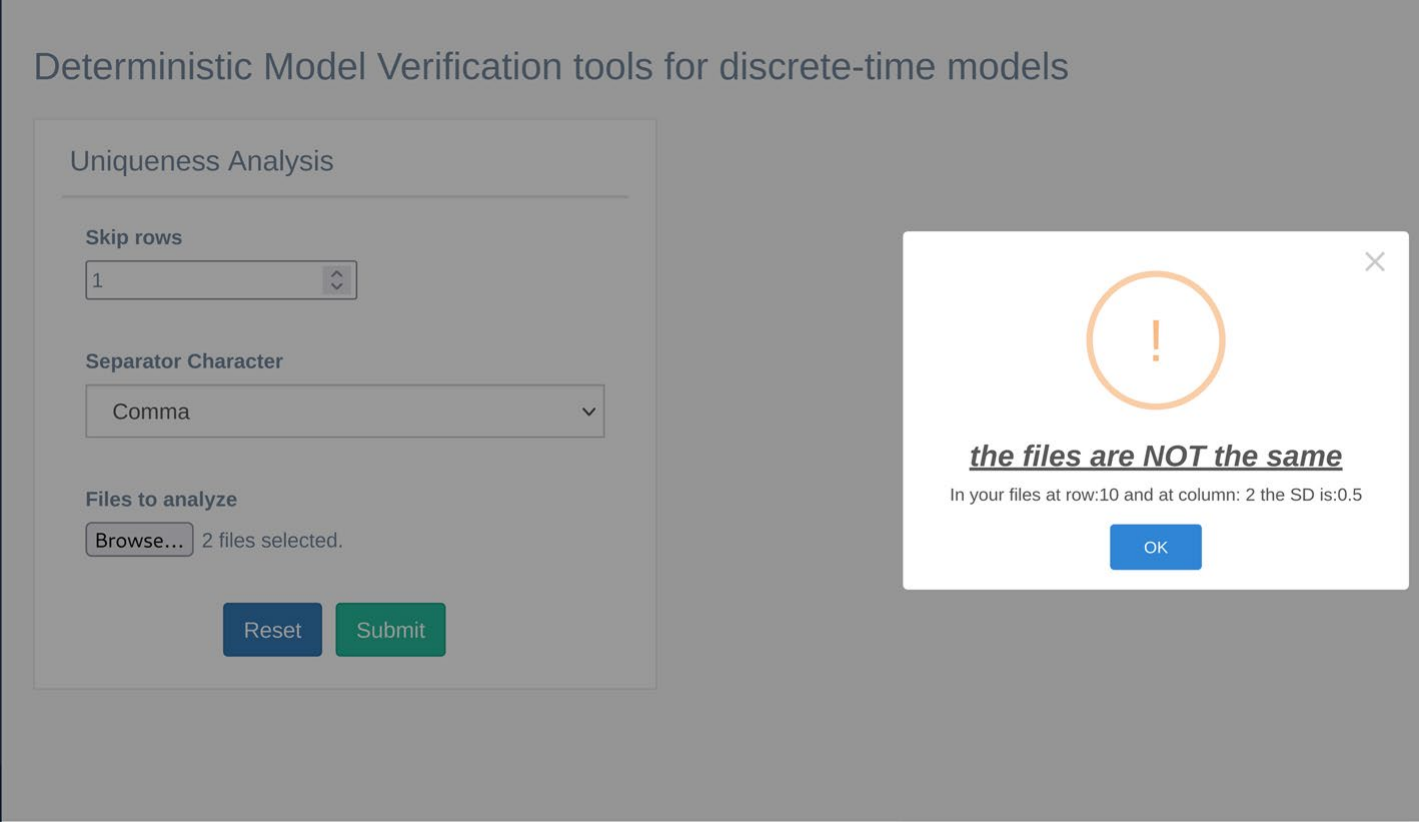
An example of the output of the uniqueness analysis, where the files are not the same. Information about the row and the column in which SD is not equal to zero is also provided

As described in the previous version of the tool [16], the user can set up the analysis parameter in the GUI (Fig. 5). In particular, the analysis needs as an input: i) “Skip rows” parameter that allows ignoring specific rows from the analysis; ii) “Separator Character” panel allows defining the correct separator character of the input files, and iii) “Files to analyze” panel allows to select the files to analyze. After clicking on the submit button, the tool calculates the mean and Standard Deviation (SD) of all the rows among all the files. If the maximum value of the previously calculated SD is not equal to 0, the files are different. In this case, the tool returns a warning message in a pop-up window, showing the row and the column of the first occurrence where the SD is different from 0 (see Fig. 6); on the other hand, MVT returns a success message in a pop-up window.

#### Uncertainty analysis

In this version of MVT, we used the scikit-optimize library to integrate the Latin Hypercube Sampling (LHS) methodology and the SALib python library for implementing the Sobol Sensitivity Analysis methodology [22–24].

**Fig. 7:**
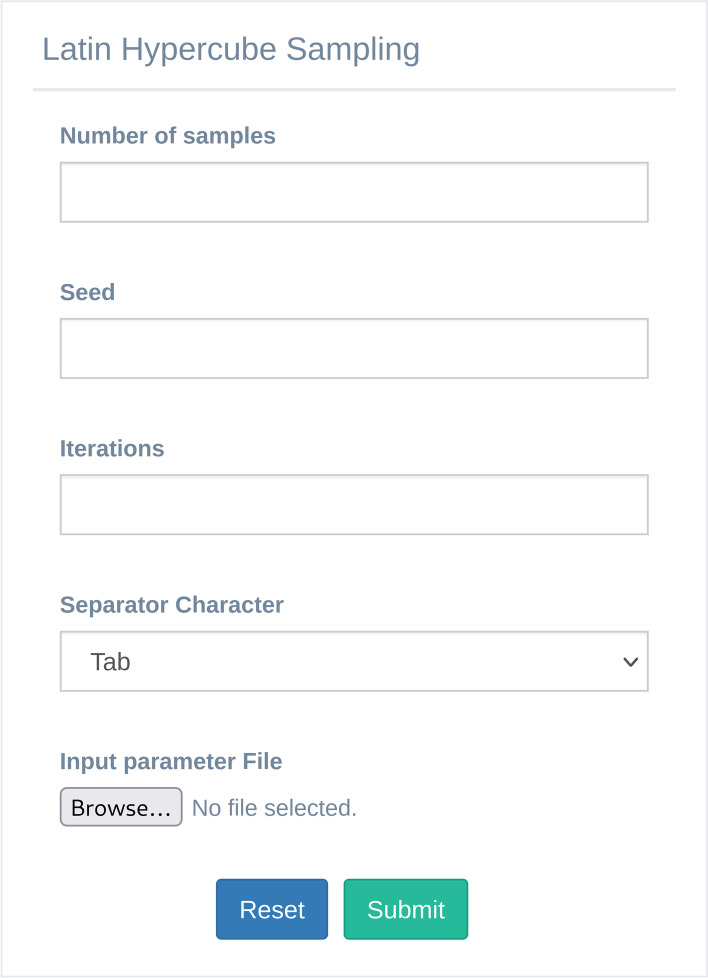
The Latin hypercube sampling analysis GUI

The user can set up the analysis parameters in the GUI for LHS presented in Fig. 7. In particular, the box allows to set: i) the “Number of samples” to define the number of samples to generate; ii) the “Seed” parameter that is used to define the random seed of the pseudo-random generator, in order of creating sample input parameter sets that can be reproduced; iv) “Iteration” field defines the number of iterations for optimizing LHS; v) “Separator Character” panel allows defining the proper separator character of the input file; vi) “Input parameter file” is the field that allows uploading a CSV file, defining the model inputs on which to perform the simulations. This file must have a header and three columns defined as follows: i) *param_name*: is the first column and represents the name of the parameter; ii) *min*: is the second column and represents the minimum value of the parameters; iii) *max*: is the third column and represents the maximum value of the parameters. In this version of MVT, the LHS tool can generate a sample set drawn from a uniform distribution of the parameters. Once the analysis is complete, it produces the LHS matrix with N rows and M columns, where N represents the number of samples and M the number of parameters. After that, the user can download the matrix and run the model on the parameter set generated.

**Fig. 8:**
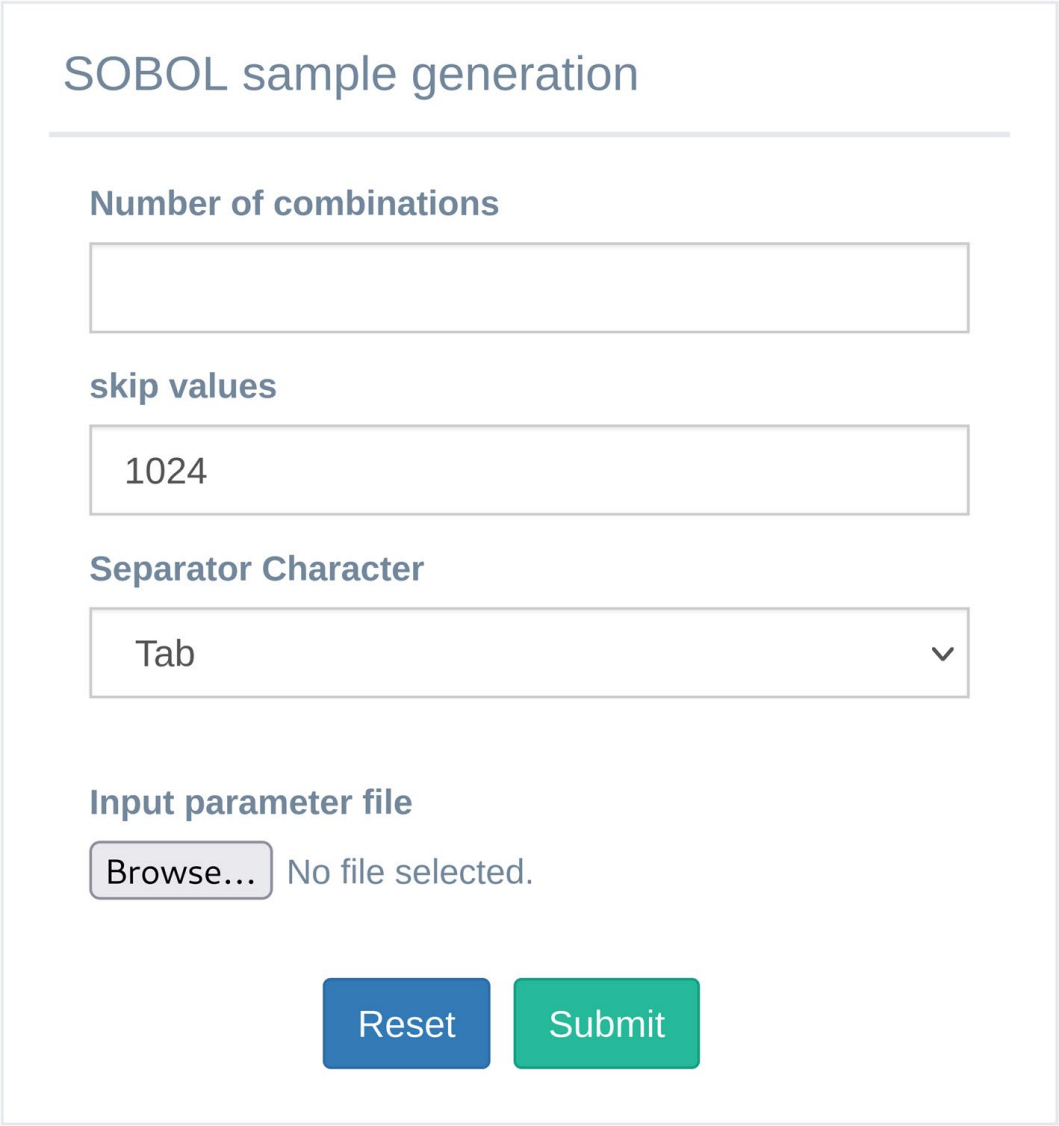
The SOBOL sample generation analysis GUI

MVT allows the user to use another algorithm to generate samples based on Saltelli methodology [20, 23] using the SALib library. Specifically, the user can use the appropriate GUI (Fig. 8) to choose the parameters for the generation samples: i) "Number of combinations" panel allows to set the number of samples to be generated; ii) "skip values," according to the SALib library, is the number of points in Sobol' sequence to skip for getting different samples. Furthermore, it is worth mentioning that this value must be an exponent to the power of 2; iii) "Separator Character" allows to define the proper separator character and, iv) "Input Parameter File" allows to choose a CSV file with the model parameters, its range values and the type of distribution to be used for the generation of each specific parameter. The CSV file must have a header with the following structure: i) *param_name*: this field represents the name of the parameters; ii) *first_value*: this value depends on the type of distribution and represents the minimum value if the value itself of the distribution field is "uniform"; otherwise, the value represents the mean; iii) *second_value*: this field also depends on the type of distribution chosen. This value represents the maximum if the value of the distribution field is "uniform"; otherwise, the value represents the standard deviation; iv) *distribution*: this field represents the type of distribution used to generate the samples for each parameter. The allowed values are: "unif", "norm" and "lognorm", which specifically represent the "uniform", "normal" and "lognormal" distribution. After clicking on the submit button, the algorithm produces a matrix having N * (2D + 2) rows. Where D is the number of parameters in input and N is the number of combinations. The number of rows in the matrix is equal to the number of samples.

#### Sensitivity analysis: Sobol analysis

**Fig. 9:**
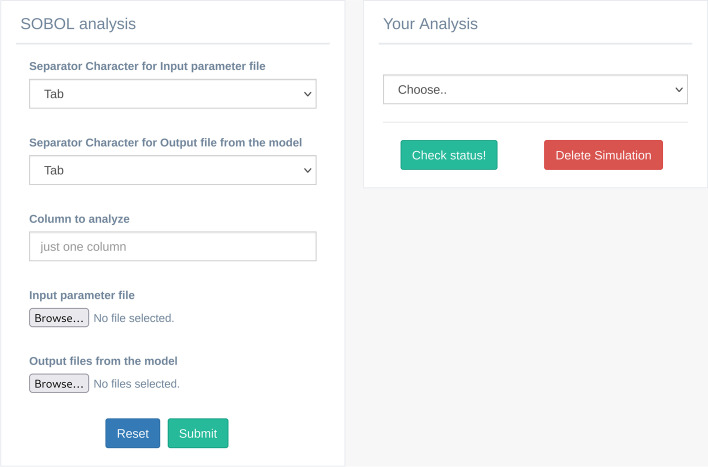
The SOBOL analysis GUI. The box on the left side represents the list of parameters necessary to perform the analysis; on the right side, the “Your Analysis” box contains the list of the completed analysis results

This analysis is used to evaluate the sensitivity of the model to the input parameters using the matrix obtained from the Sobol sample generation procedure. The user can perform this analysis using the appropriate GUI (Fig. 9), which allows to set up the following parameters: i) “Separator Character for Input parameter file” and “Separator Character for Output file from the model” allows to define the proper separator character for parameter file and for Output file which derive from the model; ii) “Column to analyze” represents the column on which to perform the analysis; iii) “Input parameter file” represents the same file used in Sobol sample generation; iv) “Output files from the model” panel is used for uploading the model outputs files, in ASCII or CSV format, without header. To perform the analysis, it is mandatory to rename the files according to a predefined scheme. The naming of the output model files should follow the following nomenclature: "0_1.csv", "0_2.csv", and so on, with the second index representing the model output obtained by the respective input row value of the Sample Generation output matrix. For example, "0_1.csv" represents the model output from the first input row, while "0_2.csv" represents the model output obtained by the second row from the Sample Generation output matrix.

#### Sensitivity analysis: PRCC analysis

**Fig. 10:**
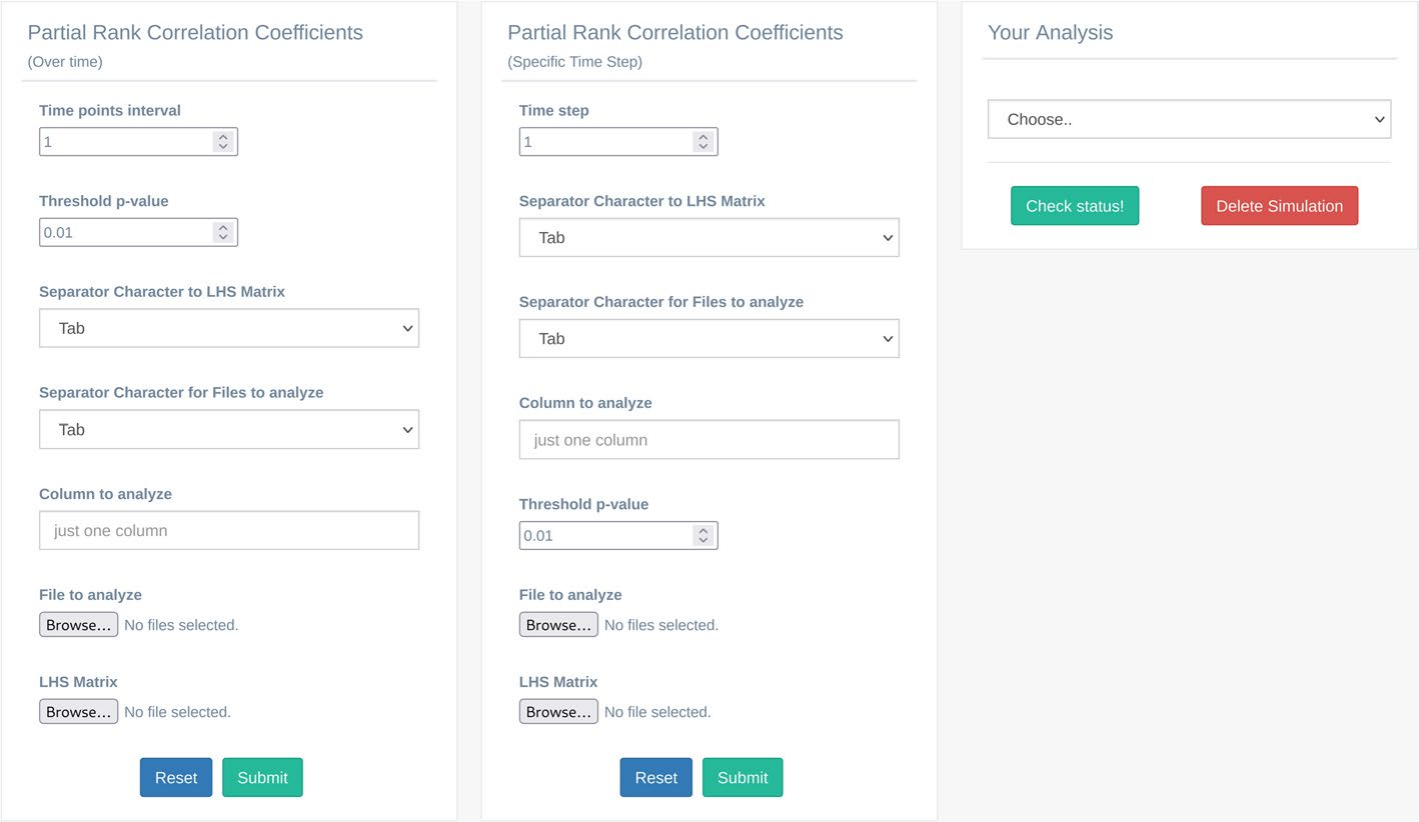
The partial rank correlation coefficients analysis GUI. The box on the left side represents the list of parameters to perform the PRCC over time analysis; while the box on the center side represents the list of parameters to perform the PRCC to a specific time step. On the right side, the “Your Analysis” box contains the list of the completed analysis results

**Fig. 11:**
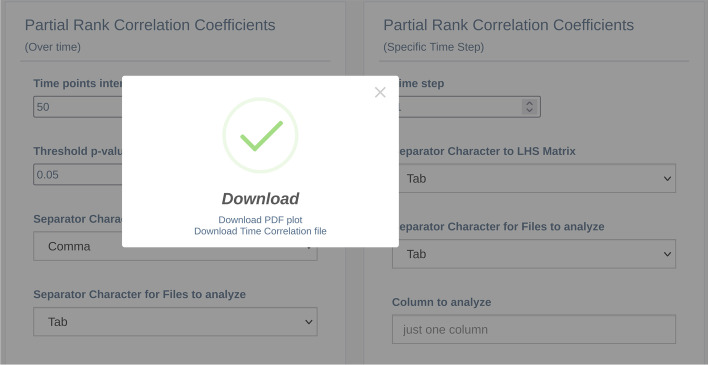
A pop-up example to download the PRCC over time plots and the time correlation files

**Fig. 12:**
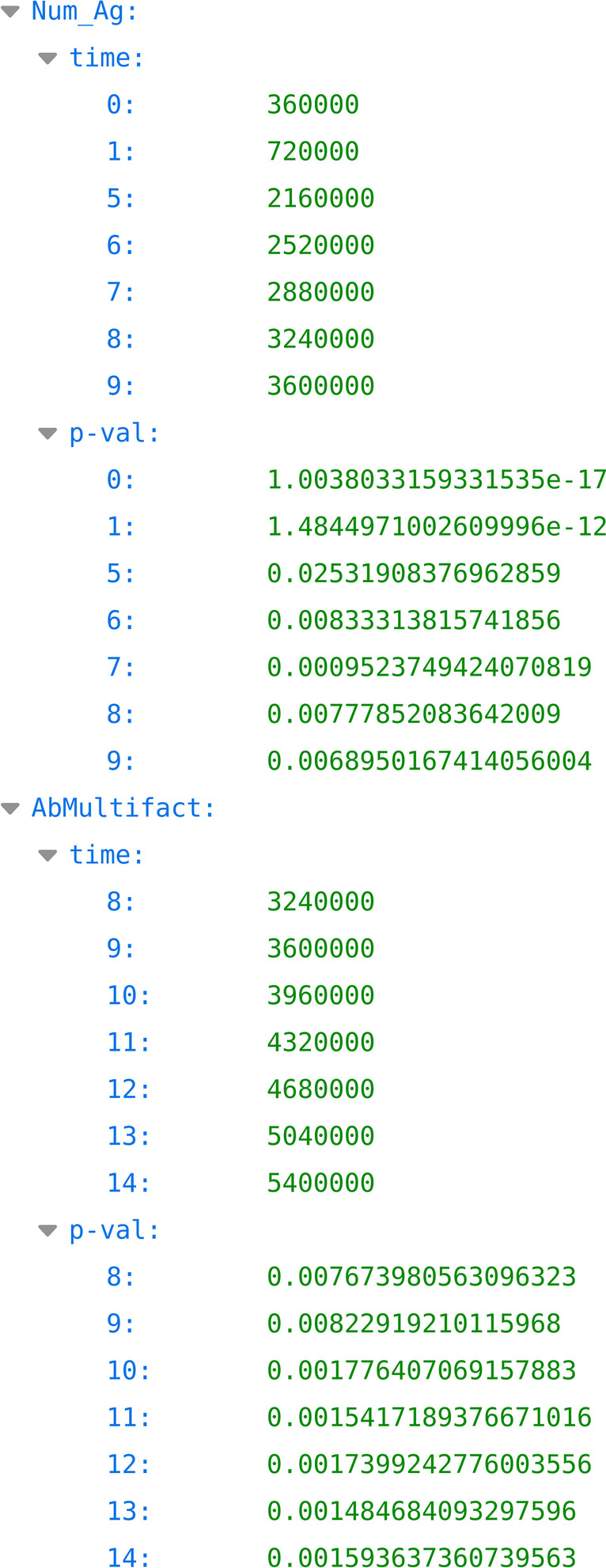
A sketch of time correlation JSON files

Once the input parameters have been generated with the LHS procedure, and the simulations have been run on such parameters, the Partial Rank Correlation Coefficient (PRCC) procedure for finalizing the LHS-PRCC analysis [25] can be executed. The user can then check the PRCC values evolution over time (PRCC_OT) and understand how the relationship between inputs and outputs evolves over time, and/or visualize the PRCC results at specific time steps (PRCC_STS); this allows to understand better the correlation among the input parameters and the output of the model. In the PRCC overtime analysis, the GUI (the box on the left side in Fig. 10) takes into account the same input parameters described for the other tools, along with the following ones: i) “Time points interval” allows to pick out the data from the “Column to analyze” for a specific time point interval selected by the user; ii) “Threshold p-value” allows the user to set up the threshold for the visualization of the level of significance. This analysis produces a pop-up (Fig. 11) that allows the user to download a pdf file containing the temporal correlation plots. Furthermore, a JSON file (Fig. 12) containing each parameter under investigation the time points in which the p-value overcomes the threshold set by the user, meaning that the correlation is significant, is also made available. MVT provides the PRCC_STS GUI (the central box of Fig. 10) to analyze the relationship between the input and output parameters at specific time points. This analysis takes the same parameters defined for the PRCC_OT but replaces the “Time point interval” parameter with “Time step.” At the end, PRCC_STS produces a pop-up window that allows users to download and visualize a scatter plot for each parameter under study. Such graphical plots are of significant importance, as they allow to graphically reveal possible non-monotonic correlations that are not usually detected by the standard PRCC procedure.

## Results and discussion

We applied the Deterministic Model Verification and Uncertainty and Sensitivity analysis techniques on UISS for the SARS-CoV-2 scenario (UISS-SARS-CoV-2). UISS-SARS-CoV-2 is the implementation of COVID-19 disease model in UISS. Hence it owns the immune system machinery originally developed inside UISS. UISS-SARS-CoV-2 was further implemented to reflect the dynamics of COVID-19 [26]. Within UISS, it is possible to change the time-step length for the simulation. In other words, it is possible to simulate with a time-step length equal to 8 h, rather than 20 min, or 5 min. Within the context of UISS-SARS-CoV-2, we run 9 simulations using a time-step length between 8 h and 5 min and with a total duration of 4 months of simulation.

**Fig. 13:**
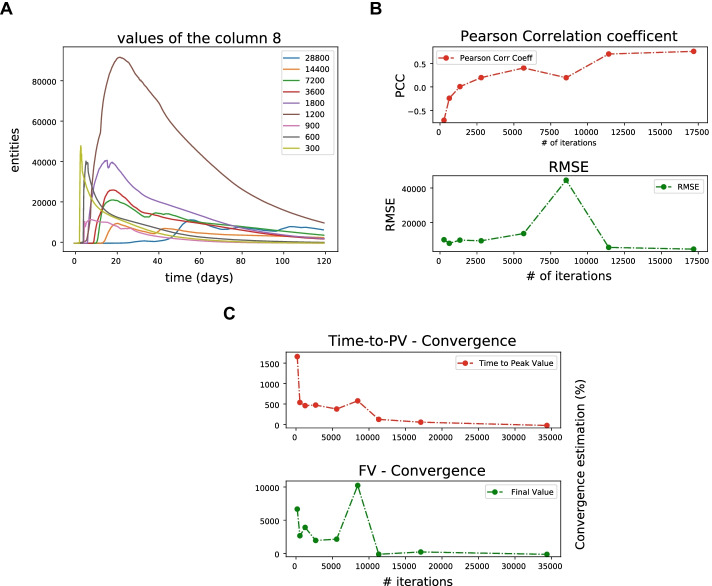
Time step convergence active TH-1 output. Panel A shows the dynamics of the output at different timestep over time. Panel B shows the Pearson Correlation Coefficient and Root Mean Square Error at different time step. Panel C shows the convergence of Time to peak value and Final value of the output

We assumed that the reference trace has a time-step length of 5 min. Then, we used the MVT to evaluate the time step convergence analysis on the active TH-1 cells. Panel A of Fig. 13 shows the specific trends of the active TH-1 cells. Panel B shows the PCC, and the RMSE computed between the reference trace and the other ones. It is worth mentioning that, at the end of the plot, the value of PCC is about 0.6, and the value of RMSE remains stable. The last step of time step convergence analysis uses the formula described above to calculate the convergence of the time-to-peak-value and the final value; the corresponding plot is shown in Panel C. The x-axis of Panel B and C shows the number of iterations, that is the number of steps to reach the end of simulation. The number of iterations depends on time-step length. According to the time step convergence analysis, the obtained results from the time-to-peak value and the final value suggest that convergence is achieved using a time-step length of 15 min, that is equal to 11,520 number of iterations.

**Fig. 14:**
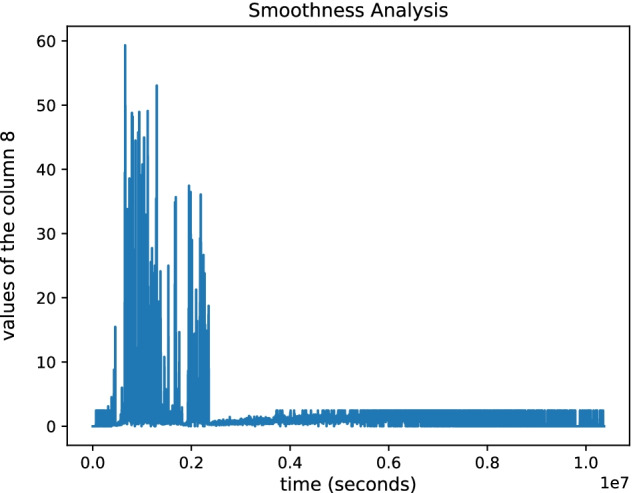
Smoothness analysis on the TH-1 Active output. The plot shows the smoothness analysis of the TH-1 Active. The data presented in the first part of the plot shows sudden peaks caused by the TH-1 response to specific antigens

**Fig. 15:**
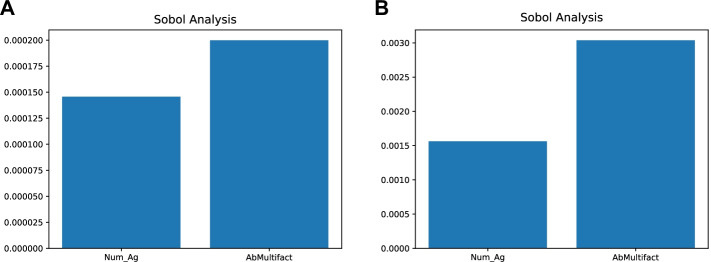
The SOBOL analysis output. Panel A and B respectively show the sensitivity of the simulator on the “Num_Ag '' and “AbMultifact '' parameters concerning TH1-active cells and IgG

For this reason, subsequent analyses will be carried out using the outputs obtained from the simulation with a time-step length of 15 min. The next step was to perform the smoothness analysis of the TH-1 Active (Fig. 14). The data presented in the first part of the plot shows sudden peaks caused by the TH-1 response to specific antigens. Then, we perform the Uniqueness analysis to check if repeated executions on the same input parameter set lead to the identical output. For this reason, we ran the UISS-SARS-CoV-2 three times with the same set of input parameters obtaining identical outputs. Sobol is the first technique applied to carry out the Uncertainty and Sensitivity analysis of the UISS-SARS-CoV-2. We chose to evaluate the sensitivity of the simulator by varying the values of two parameters: i) Num_Ag, the number of inoculated antigens within the range; ii) AbMultifact, the number of antibodies secreted by plasma B cells, within the range. It is important to note that each parameter was sampled using a uniform distribution. Then, through the GUI of Sobol sample generation, we chose to set the 'number of combinations' parameter equal to 16 in order to generate 96 samples. After running the simulations, we chose to analyze the relationship between the input set and active TH-1 cells and IgG, respectively. Panel A in Fig. 15 shows the correlation relationship between the two input parameters (Num_Ag and AbMultifact levels) and active TH-1 cells levels, while Panel B shows the sensitivity result obtained for the IgG values. These figures make it possible to highlight how, inside our model, the two parameters differently affect the selected outputs. It is important to remember that the greater the sensitivity indices are, the more critical parameters are for the model output. Therefore, we can observe how the dynamics of IgG antibodies are more sensitive to the variation of the number of inoculated antigensdue to the low y value in panel B, while AbMultifact mainly affects the dynamics of active TH-1.

**Fig. 16:**
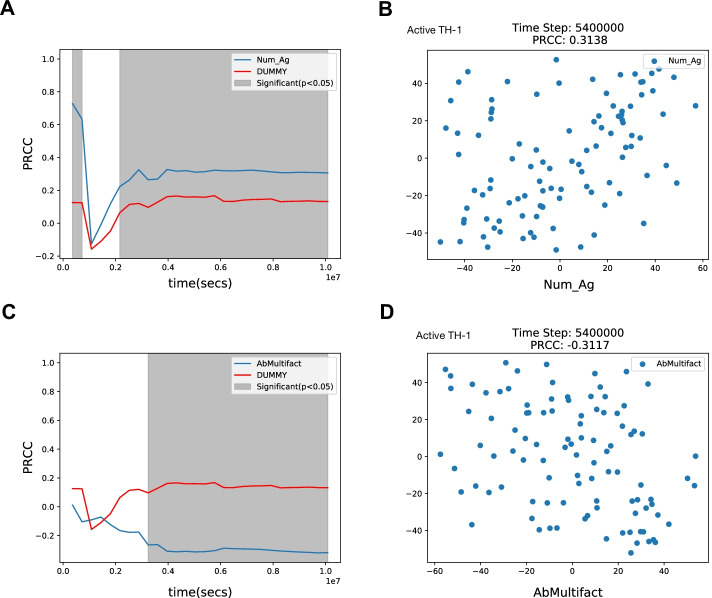
The output of PRCC_OT And PRCC_STS. Panel A and B respectively show the sensitivity of the simulator on the “Num_Ag” over time and at a specific time step. Panel C and D respectively show the sensitivity of the simulator on the “AbMultifact” over time and at a specific time step. The scatter plots, Panel B and D, depict the influence of the Num_Ag and AbMultifact variables (input, x-axis) on the selected output value (y-axis), respectively

LHS-PRCC is the second technique applied to perform the Uncertainty and Sensitivity analysis of the model. The parameters and the type of distribution used here are the same as those taken into account in the previous analysis. In order to generate the LHS matrix, the parameters of GUI were set as follows: i) 'number of samples’ equals 96; ii) ‘Seed’ parameter equal 2021; iii) ‘Iterations’ equal 1000. After that, the simulation was run on the LHS matrix. After that, we chose to apply the PRCC_OT and PRCC_STS analysis to analyze the relationship between parameters set and TH-1 active cells and IgG. The plots of PRCC_OT are shown on panels A and C of Fig. 16. Both panels show a dummy curve (red line), it does not affect the model in any other way, but it is useful for comparing parameters that have an effect on the model output. Panel A shows a strong correlation at the start of simulations between Num_Ag and active TH-1 (highlighted in gray), which then turns into a weak correlation between 0.1*10^7^ s and 0.2*10^7^ s and then turns again into a strong correlation until the end of the simulations. Panel C shows a weak correlation at the start of simulations between AbMultifact and the levels of active TH-1 cells, which then turns into a strong correlation until the end of the simulations. After that, we ran the PRCC_STS analysis at the specific time step of 5,400,000 s, obtaining the plots shown in panels B and D of Fig. 16. These scatter plots, depict the influence of the Num_Ag and AbMultifact variables (input, x-axis) on the selected output value (y-axis), respectively. Both input and output values are represented as ranked values to remove any non-linear relationship. Scatter plots may be useful to visually detect the presence of non-monotonic relationships that are not usually shown by the PRCC value alone. In this case Fig. 16, Panel B, shows a weak positive relationship at time-step 5,400,000 between the Num_Ag variable and the output value (PRCC: 0.3128). This can be seen by observing that the distribution of the points vaguely approximates an increasing straight line. Conversely in Fig. 16, Panel D, a weak negative relationship between the AbMultifact variable and the output value (PRCC: 0.3128) holds, with the points distribution that vaguely approximates a decreasing straight line.

## Conclusions

Mechanistic agent-based models are increasingly employed for developing in silico trials solutions for medicinal products. Consequently, to lower barriers in their regulatory acceptance, the assessing of their credibility is mandatory. Formal methodologies for agent-based models verification should be developed and widely adopted. The described approach proposes a set of automatic tools that help formally verifying the deterministic part of mechanistic agent-based models. This allows researchers and practitioners to easily perform verification steps to prove ABM robustness and correctness that provide strong evidences for further regulatory requirements. As ABMs usually own a stochastic component, statistical consistency and minimum sample size determination need to be also addressed. We are dealing with this issue and we will expand actual modeling verification framework in due course.

### Availability and requirements

Project name: Model Verification Tools

Project home page: https://github.com/COMBINE-Group/docker_verify

Operating system(s): Platform independent

Programming language: Python

Other requirements: Docker

Any restrictions to use by non-academics: not applicable.

## Data Availability

The main computational framework is fully described in the paper. The UISS-SARS-CoV-2 framework used for this research is available at: https://combine.dmi.unict.it/UISS-COVID19/. The MVT tool is available at: https://github.com/COMBINE-Group/docker_verify.

## Abbreviations

VV&UQ: Uncertainty quantification
ISTs: In silico trials
ABMs: Agent-based modeling
MVT: Model verification tools
FITA: Fixed increment time advance
LHS-PRCC: Latin hypercube sampling-partial rank correlation coefficient (LHS-PRCC)
LHS: Latin hypercube sampling
PRCC: Partial rank correlation coefficient
GUI: Graphic user interface
PV: Peak value
FV: Final value
PCC: Pearson correlation coefficient
RMSE: Root mean square error
SD: Standard deviation
UISS: Universal immune system simulator

## Footnotes

1 https://github.com/COMBINE-Group/docker_verify

2 https://www.djangoproject.com/

3 Django (Version 1.5) [Computer Software]. (2013). Retrieved from https://www.djangoproject.com/
